# Hydatid Cyst of Thyroid Gland: A Case Report

**DOI:** 10.1002/ccr3.70337

**Published:** 2025-04-09

**Authors:** Prashant Ghimire, Sagar Rana Magar, Bishal Panthi, Prem Bahadur Maharjan, Intjar Khan, Neeraj Thapa, Sujan Paudel, Siddhartha Karn, Prajjwol Luitel

**Affiliations:** ^1^ Universal College of Medical Sciences Bhairahawa Nepal; ^2^ Tribhuvan University Institute of Medicine Kathmandu Nepal; ^3^ Department of Pathology Tribhuvan University Teaching Hospital Kathmandu Nepal; ^4^ Kathmandu University Dhulikhel Nepal; ^5^ Nepal Medical College Teaching Hospital Kathmandu Nepal; ^6^ Institute of Medicine Kathmandu Nepal

**Keywords:** anti‐parasitic drug, FNAC, hydatid cyst, lobectomy, thyroid gland

## Abstract

Hydatid cysts in the thyroid gland are extremely rare, even in endemic areas. A 64‐year‐old male presented with a painless swelling on the left side of the thyroid swelling for 2 years. Ultrasonography revealed a large multiloculated anechoic lesion while fine needle aspiration cytology yielded clear watery to granular fluid containing hooklets, protoscolioces, laminated membrane, identifiable on both Giemsa stained and unstained slides. Further tests confirmed positive serology for *Echinococcus*, and computed tomography (CT) scan showed no such cysts in other organs. The patient was treated successfully with a lobectomy without signs of recurrence in 1 year. Although primary hydatid cysts of the thyroid are rare, even in endemic areas, they should be considered as a differential diagnosis when evaluating thyroid nodules in these regions. Fine needle aspiration cytology (FNAC) can confirm the diagnosis. However, clinicians must take care to avoid anaphylactic reactions. The use of FNAC in hydatid disease is debatable as it may cause anaphylactic reaction, but in cases with doubtful diagnosis, it can serve as a confirmatory tool. The condition can be managed successfully with lobectomy.

**Summary**

- Ultrasonography (USG) and computed tomography (CT) are important modalities for detecting hydatid cysts.
- Fine needle aspiration cytology (FNAC) can confirm the diagnosis when doubtful.
- Despite their rarity in the thyroid, primary hydatid cysts should be considered as differential diagnosis for thyroid swellings, even in areas.
- Surgical resection remains the treatment of choice. FNAC, hydatid cyst, thyroid gland, lobectomy, anti‐parasitic drug

## 1 Introduction

Hydatid disease is a significant health concern in many parts of the world, including the Middle East, Southeast Asia, Mediterranean regions, and South America. It accounts for approximately 871,000 disability‐adjusted life years (DALYs) annually [1].

Those regions where livestock is commonly raised, such as Southeast Asia, have an incidence of 0.8 cases per 100,000 people, according to the World Health Organization (WHO) [2]. Like other developing countries, Nepal also has a high incidence of echinococcosis, primarily due to poor hygiene and sanitation practices [3].

Hydatid cyst is a zoonotic infection caused by the larval form of the tapeworm *Echinococcus granulosus* [2]. It is most commonly found in the liver (50%–77%), followed by the lungs (15%–47%), spleen (0.5%–8%), and kidneys (2%–4%) [4]. The prevalence of hydatid cysts in the thyroid gland is exceedingly rare, with a reported prevalence ranging from 0% to 3.4% [4]. It is rare even in endemic countries [5]. It is generally reported to be diagnosed only after surgical excision [5].

FNAC is widely recognized as the initial step in evaluating thyroid swelling. However, in patients with thyroid hydatid cysts, it poses a risk of anaphylactic shock, widespread cyst dissemination, and severe local inflammation complicating the surgery [6].

Surgical excision is the treatment of choice for primary hydatid cyst of thyroid. Anti‐parasitic medication therapy with albendazole or mebendazole is recommended for patients in whom surgery cannot be done [7].

In line with CARE guidelines, we present a rare case of primary hydatid cyst in the thyroid gland diagnosed by FNAC and treated successfully with lobectomy [8].

## 2 Case History/Examination

A 64‐year‐old male cattle farmer presented to the out‐patient department with the complaint of asymptomatic neck swelling for 2 years, with its size being static since last 1 year. He reported no associated change in voice, dysphagia, dyspnea, fever, night sweats, or weight loss. On physical examination, a well‐defined nodular swelling measuring 7 cm × 4 cm was present on the anterior neck on the left side. The mass moved with swallowing but not with protrusion of the tongue; the overlying skin was intact, and the mass was non‐tender.

## 3 Methods

Preoperative diagnosis of a hydatid cyst of thyroid origin can be challenging, as it can mimic tumors, abscesses, and other space‐occupying thyroid lesions such as thyroglossal cysts and branchial cleft cysts [4, 9].

The laboratory and pathological findings of this patient can be presented Tables 1 and 2 respectively.

**TABLE 1:** Laboratory findings.

Laboratory tests	Findings
Complete blood count (CBC)	
WBC	7800/mm^3^
2 RBC	4,800,000/mm^3^
3 Hb	15 g/dL
4 Hematocrit	42%
Thyroid function test (TFT)	
T3	100 ng/dL
2 T4	8.6 mcg/dl
3 TSH	2.785 mU/L
Renal function test (RFT)	
Serum creatinine	68 μmol/L
2 Serum urea	4.6 mmol/L
3 Serum Na+	141 mEq/L
4 Serum K+	4.3 mEq/L
5 Urine R/E	WBC: 0–1, RBC nil, Albumin nil
Random blood sugar (RBS)	4.9 mmol/L
Erythrocyte sedimentation rate (ESR)	11 mm/h
C‐reactive protein (CRP)	0.25 mg/dL
Enzyme linked immunosorbent assay (ELISA)	Positive for *Echinococcus granulosus*
Serum IgG level	13 Net Titer Units (NTU)

**TABLE 2:** Pathological findings.

Pathological tests	Findings
FNAC	Yielded 18 mL of clear watery to granular fluid
Cytocentrifuged stained and unstained smear	Multiple protoscolices with rostellum containing rows of refractile hooklets, numerous free hooklets having claw‐like appearance with a pointed end and a bifid end, and fragmented laminated membranes of ectocyst and germinative layer (Figure 2)

**FIGURE 1:**
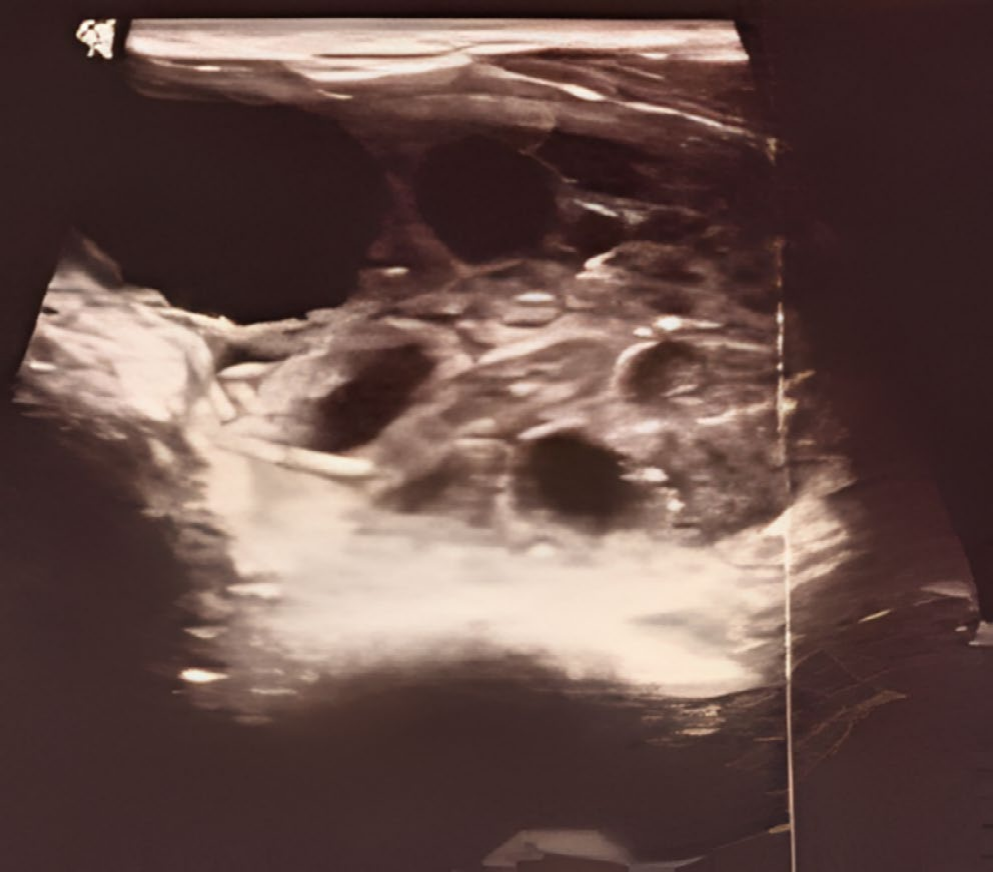
Multiloculated anechoic lesion on sonography.

**FIGURE 2:**
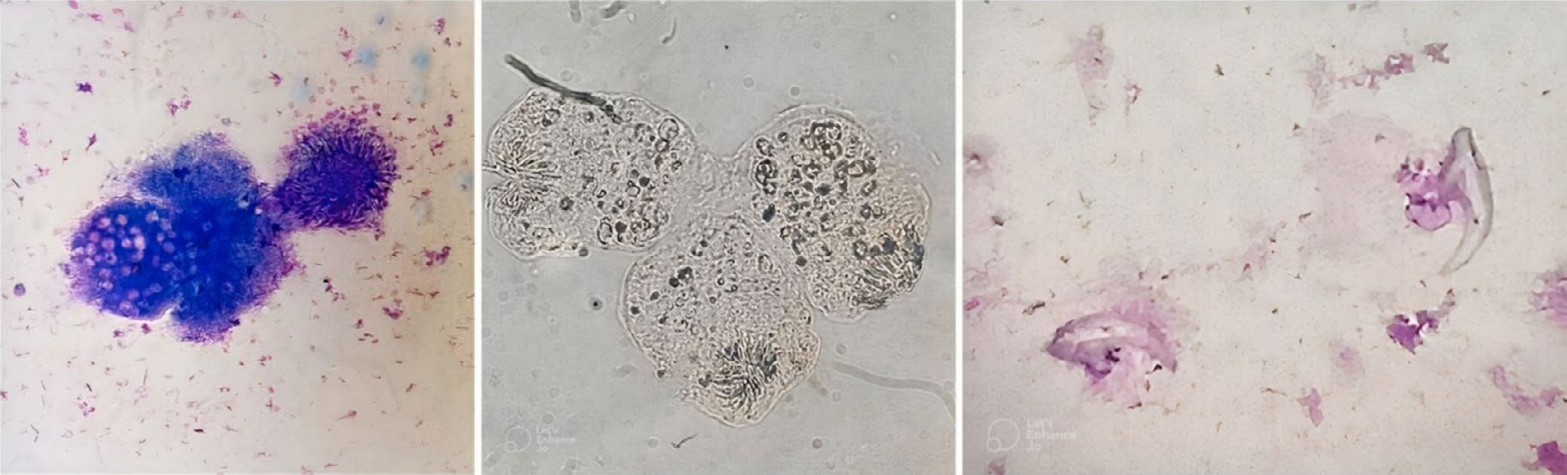
Fine needle aspiration cytology, Giemsa‐stained smear(left), unstained smear showing scolices with radially arranged hooklets (middle) and suckers and detached hooklets with pointed one end and bifid other end (right).

Ultrasonography (USG) of the neck revealed a large multiloculated heterogeneous, anechoic lesion measuring 8.1 cm × 4 cm × 5 cm in the left thyroid lobe and was designated a TI‐RADS (Thyroid Imaging Reporting and Data System)‐3 lesion (Figure 1). The right lobe and surrounding lymph nodes appeared normal. Computed tomography (CT) scan showed that the lesion was limited to the thyroid gland. The patient denied any previous history of hydatid disease, and no similar symptoms were reported in his family. With the help of FNAC (Figure 2), the final diagnosis of the primary hydatid cyst in the thyroid gland was made.

**FIGURE 3:**
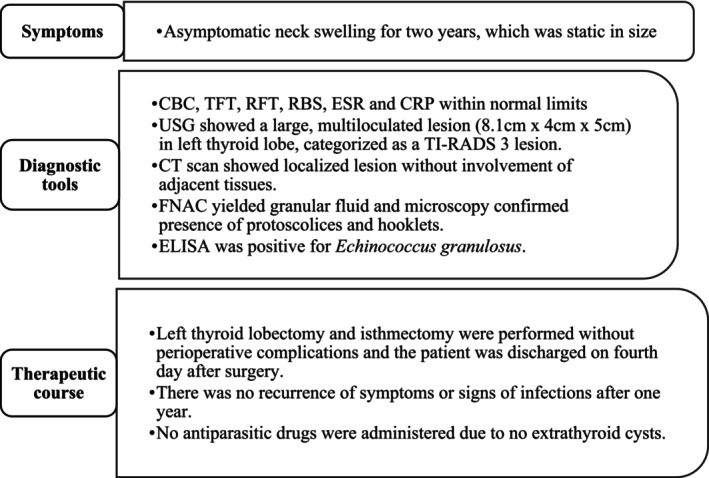
Timeline of symptoms, diagnostic tools, and therapeutic course.

The patient underwent left thyroid lobectomy and isthmectomy under general anesthesia. The excised tissue sample was sent for histopathological evaluation. The perioperative course was uneventful with no change in voice, swallowing difficulty, surgical site infections, and symptoms of hypocalcemia. The patient was discharged on the fourth postoperative day with prescriptions of levothyroxine, calcium, and calcitriol. Histopathology revealed an outer cuticular membrane and an inner germinal layer surrounded by a pericyst layer confirming a hydatid cyst. Anti‐parasitic drugs were not prescribed since there were no hydatid cysts in other sites. During follow‐up at 1 year, he had no recurrence of symptoms or signs of *Echinococcus* infection. The following timeline (Figure 3) summarizes the symptoms, diagnostic, and therapeutic course of this patient:

## 4 Conclusion and Results

Surgical intervention was successfully carried out in our case and no anti‐parasitic drugs were administered since there were no postsurgical complications such as infections at the surgical site and no evidence of extrathyroid hydatid cysts. During follow‐up at 1 year, he had no recurrence of symptoms or signs of *Echinococcus* infection and the CT scan confirmed that there was no recurrence of the hydatid cyst.

## 5 Discussion

The first reported case of thyroid involvement with hydatid cyst was in 1946 [4]. While there have been some reports of thyroid hydatid cysts from endemic regions, to our knowledge, there have been none from South Asia.

Hydatid cyst of thyroid can develop either primarily (affecting only the thyroid) or secondarily (involving multiple organs). The possible pathogenesis is emerging from the oncosphere larval stage in the gastrointestinal tract and entering the bloodstream, bypassing or passing through the liver and eventually reaching the thyroid gland [5]. Humans become accidental hosts by accidentally ingesting contaminated food or water or through direct contact with definitive hosts [10]. In our case, the patient had a history of exposure to cattle and sheep similar to that in previous cases.

The symptoms of a hydatid cyst in the thyroid depend upon the size and adherence of the lesion to surrounding structures, with most of the patients being asymptomatic, except for a non‐progressive swelling, as seen in our case. The slow growing pattern may explain the lack of symptoms in the patient with clinical dormancy for several years [11]. However, patients may experience difficulty breathing, swallowing, hoarseness, anaphylaxis, formation of pyogenic abscess, and cystotracheal fistula secondary to erosion of the tracheal wall by cyst [4].

USG is a routine imaging tool for visualizing cysts and daughter vesicles. CT/MRI is used to assess the location of the cyst in relation to its surrounding structures [4].

Despite having limited sensitivity and specificity in primary thyroid hydatid cyst disease, immunological methods like indirect hemagglutination, latex agglutination, ELISA, and immune electrophoresis may assist in the diagnosis when it remains unclear [12].

The role of FNAC in preoperative diagnosis of thyroidal hydatid cyst remains controversial despite its higher sensitivity and specificity pertaining to the high risk of anaphylactic reactions and the potential dissemination of cystic elements, which can cause inflammation and complicate surgery [4].

In our case, the patient did not experience any immediate or late reactions following the USG‐guided FNAC.

An ultrasound scan of the neck was performed but was non‐conclusive, while FNAC confirmed the diagnosis of a hydatid cyst in the thyroid, specifically identifying a unilateral hydatid cyst in the left lobe of the thyroid gland. To the best of our knowledge, only one other case of a thyroid hydatid cyst has been diagnosed through FNAC before the surgery, as in our case [6].

While postsurgical histopathology is considered the gold standard for diagnosis, an integrated approach that includes patient history, clinical examination, imaging tools like USG, CT scan, magnetic resonance imaging (MRI), scintigraphy, serological examination, and FNAC is vital for accurate diagnosis [4].

Surgical resection is the preferred treatment option. In cases where surgery is contraindicated, anti‐parasitic drugs like mebendazole or albendazole can be administered as postsurgical adjuvant chemotherapy to prevent recurrence. Follow‐up after surgery is recommended at 3 months, 6 months, and 1 year of surgical operation [5, 7]. In this case, the patient's condition significantly improved post‐surgery without needing anti‐parasitic medications, as there were no postsurgical complications.

## Author Contributions

**Prashant Ghimire:** conceptualization, data curation, formal analysis, funding acquisition, investigation, methodology, project administration, resources, software, supervision, validation, visualization, writing – original draft. **Sagar Rana Magar:** conceptualization, data curation, formal analysis, funding acquisition, investigation, methodology, project administration, resources, software, supervision, validation, visualization, writing – original draft, writing – review and editing. **Bishal Panthi:** conceptualization, data curation, formal analysis, funding acquisition, investigation, methodology, project administration, resources, software, supervision, validation, visualization, writing – original draft. **Prem Bahadur Maharjan:** conceptualization, data curation, formal analysis, funding acquisition, investigation, methodology, project administration, resources, software, supervision, validation, visualization, writing – original draft. **Intjar Khan:** conceptualization, data curation, formal analysis, funding acquisition, investigation, methodology, project administration, resources, software, supervision, validation, visualization, writing – original draft. **Neeraj Thapa:** conceptualization, data curation, formal analysis, funding acquisition, investigation, methodology, project administration, resources, software, supervision, validation, visualization, writing – original draft. **Sujan Paudel:** conceptualization, data curation, formal analysis, funding acquisition, investigation, methodology, project administration, resources, software, supervision, validation, visualization, writing – original draft, writing – review and editing. **Siddhartha Karn:** conceptualization, data curation, formal analysis, funding acquisition, investigation, methodology, project administration, resources, software, supervision, validation, visualization, writing – original draft, writing – review and editing. **Prajjwol Luitel:** conceptualization, data curation, formal analysis, funding acquisition, investigation, methodology, project administration, resources, software, supervision, validation, visualization, writing – original draft, writing – review and editing.

## Ethics Statement

The authors have nothing to report.

## Consent

Written informed consent was obtained from the patient to publish this report in accordance with the journal's patient consent policy.

## Conflicts of Interest

The authors declare no conflicts of interest.

## Guarantor

Prajjwol Luitel is the guarantor.

## Data Availability

Data supporting the conclusions of this report are contained within the report. Additional non‐relevant patient data are protected under patient privacy regulations and policies.
